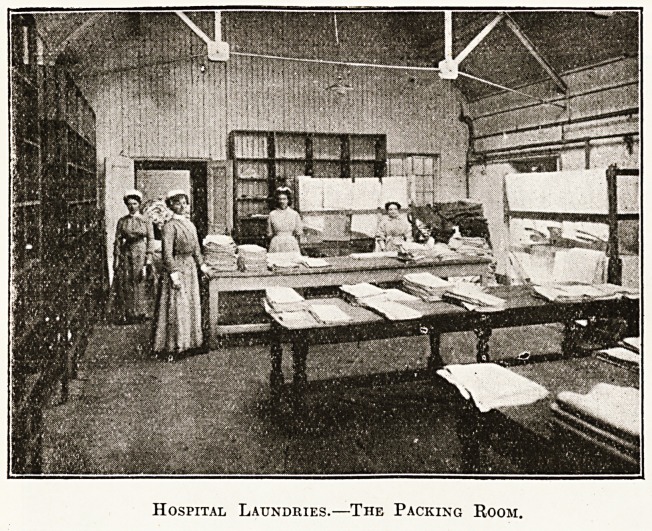# A Provincial Laundry Experience

**Published:** 1912-11-16

**Authors:** 


### A Provincial Laundry Experience.

BY THE HOUSE GOVERNOR AND SECRETARY.

Hospital laundries and public laundries differ very little from each other. Their processes and their equipment are to all intents and purposes the same. They each have collecting rooms, sorting rooms, washing rooms, ironing rooms, packing rooms, etc. A hospital laundry does not, however, undertake the same amount of fine work as a public laundry; its pieces are large—sheets, counterpanes, draw sheets, blankets, etc., form the bulk of its work, and a great deal of this work is infected and foul to a degree unknown in public laundries. If, then, the work required by a hospital is so similar to the work done by a public laundry, it may be asked why should the hospitals go to the expense and trouble of putting up a building and plant, and collecting a staff to do work that can equally well be done by public laundries, probably at a lower cost? The answer to such a question is, I think, not difficult. Even if the reduction in cost be conceded (in any case it would not amount to very much), the saving in the long run would be more than made up by the greater destruction of the goods, for that the public laundries do destroy the goods will be allowed by all, I think, except the managers of the laundries themselves. The wear and tear on hospital sheets is quite enough, without adding the ordeal of a public laundry. But to leave the question of cost altogether. The primary object of a hospital laundry should be to get the articles absolutely clean, and before they are sent to the wards, absolutely dry; as clean, that is, as soap and water can make them, and so dry that there is no danger in putting patients into the sheets as they come from the laundry. If this cleanliness and dryness require more soap and water and more heat than the commercial laundries find necessary, then the extra expense must be cheerfully faced. This does not in the least mean that there should be no check whatever upon the soap or water bill; but it does mean that economy should not be carried to the extent of saving soap and water at the expense of cleanliness. Hospital sheets are not like household sheets. A moment's reflection will satisfy the inquirer on this point. Death and disease are the exception and not the rule in private houses; the reverse is the case in hospitals. Nowadays, the excuse would not be acceptable that a patient who lies on an imperfectly washed sheet is in blissful ignorance of the fact. Whether a patient knows it or not the sheet on which he lies must be sheet and nothing else; there must be nothing of the previous patient about it. But besides being clean, hospital linen must be dry; everything must be sent back to the wards bone dry. Sheets, blankets, etc., do not come back from the public laundries bone dry. They have to be aired before they can be put on the beds.

The prompt way in which a laundry on the premises can deal with the soiled linen and the certainty that the goods will be returned on the proper day enables each department in a large hospital to be run with a smaller stock of linen, and this fact must be taken into consideration in coming to any determination as to the merits or demerits of a hospital laundry as compared with a public laundry. And in making such a comparison it must not be forgotten that a hospital laundry cannot be run on commercial lines. In the first place, the conditions of labour must be the best that can be made. It would be ludicrous for a hospital, which is not only a place where the sick are cured, but which is also a centre from which knowledge ought to be diffused, to subject its workers to unhealthy conditions, to help to create, in fact, its own patients. The rooms must be large and airy. The fumes of gas, if gas-irons be used, must be got rid of. The feverish pace at which one sees girls working in public laundries cannot be tolerated. Managers of commercial laundries will, naturally, not agree; they will probably tell you that the laundry girl is the healthiest girl in the works, and so on. All that I can say is that I have seen a good many public laundries, and I would not invest my money in any unless the conditions were better than they are in those I have seen. However, I do not wish to labour this point. I would only repeat that I consider a laundry to be a necessary adjunct to a hospital of, at any rate, more than one hundred beds, whether it be an expensive one or not.

In building a hospital laundry space is of importance. In the provincial towns, where sites are, generally speaking, easier to obtain than in London, there is no excuse for cramping the laundry. Hospitals do not decrease in size, nor do they remain stationary, and even although the number of their beds may not go up, yet the demands on the laundry are constantly increasing. New fashions create new demands. The modern garb worn by the hospital surgeon in the theatre, his house surgeon, the nurses, and the visiting doctors from other schools, who come to see operative work, has added a very considerable amount of extra washing to the laundry. To leave no room for expansion is very questionable wisdom.

The following description of a laundry of a provincial hospital of about 450 beds may be taken as typical: The position of the laundry is not unimportant. Distances in a modern hospital are very great, and it is wise to place the laundry as conveniently as possible—I mean taking the collection and delivery of the linen in view; and here it may be as well to say that laundries are often placed immediately adjacent to the engine house, with the consequence that the engine-house chimney pours its smoke over the roof of the laundry and the coals that are delivered in the engine-house yard scatter their dust through the laundry windows. In a large town black smuts are unavoidable, but there is no reason to place a laundry just where it gets the full benefit of the engine-house chimney and the engine-house yard. The soiled linen, when it is collected from the wards, is taken to a sorting room, which should be about 30 feet square, and is there sorted into bins ranged round the walls. These bins should be of large size, large enough to hold in one 600 sheets, in another 200 blankets, a third 500 pillow slips, a fourth 800 towels, and so on. It is wise to have plenty of room here, and too many rather than too few bins. A hospital wash is bi-weekly, that is to say, Monday's, Tuesday's, and Wednesday's soiled linen, etc., constitute one wash, and Thursday's, Friday's, Saturday's, and Sunday's the other wash. There is, in point of fact, a little overlapping, some of Thursday's soiled linen being brought down before the sorting room is quite clear of the soiled linen from the previous three days. To avoid mixing the two washes it is convenient to have a few spare bins. Besides the bi-weekly wash, soiled linen from the theatres and out-patients is sorted, tanked, washed, and dried, and sent out daily, and this is an additional reason why space is valuable in the sorting room. The stained and foul linen is not sorted into the bins, but is sorted direct into the tanks. These tanks, of which there should be four or five, are conveniently placed in the washhouse. Glazed earthenware tanks, though expensive, are on the whole the most satisfactory, while steeping tanks made of teak, whether they are clean or not, do not give the impression of cleanliness. By sorting the stained and soiled linen first into bags, and then putting the bags into the tanks, one tank can, of course, be made to do for

Hospital Laundries.—The Collecting Room.

a variety of articles, but three or four steeping tanks in a large washhouse is not an extravagant number. These tanks contain ordinary cold water with so much disinfectant added.

For the ordinary theatre linen that is washed every day a hasty steeping is all that can be given; but for the skin and isolation linen a little longer steeping is necessary. It is advisable to steep as long as three days and change the water, the skin linen being steeped in very strong soda-water as well as in the disinfectant. The washhouse, which should adjoin the sorting room, should be of large size and well ventilated. The floors of concrete should be well drained. Glazed bricks should form the walls of these as well as the other rooms in the hospital laundry.

The machinery in a washhouse can be arranged in a variety of ways. As good a plan as any is to put it round the walls, not so close as to make access to the back parts difficult, nor so far into the room as to waste space. The equipment of such a washhouse should be not less than three rotary washers, two large and one small; the smaller - sized one is easier for a woman to work. The two larger can be looked after by the washman. It is of great importance to have the access doors to these rotary washers of large size. Adjoining the washers there should be two metal tanks, each holding about twenty-five gallons, with steam and water connection for boiling soap, soda, etc. To dry the linen as it comes from the washers there should be two hydro extractors, or, if small-sized hydro extractors be used, three. Hydro extractors have to be packed carefully, and it is more difficult to pack a large hydro extractor than a small one. If a hydro extractor of a large size is used it is possible to divide the cage into equal compartments, thus not only facilitating even packing, but preventing what actually does occur in hydro extractors of large size, the tearing of the fabrics. A very great strain indeed is put upon the linen when once the cage has reached a high rate of speed. A starch boiler, a hand starching machine, a starch dasher, a steam boiling trough, and four or five hand washing troughs complete the ordinary equipment of the washhouse. An old-fashioned but a very useful addition to the equipment is a box mangle. All this machinery can be driven by two sets of shafting, and, if electricity is used, two motors will be required. A good water-softening apparatus is a valuable addition to the laundry. The various appliances at present on the market cannot be said to be altogether satisfactory, and the cost of installation is very considerable.

Hospital Laundries.—The Washhouse.

For the washing of blankets a certain amount of soft water may be simply and cheaply obtained by filling a large wooden vat with water, which by the addition of a small quantity of soda and lime in proper proportions will, if allowed to stand all night, become softened. Adjoining the washhouse is the ironing room, with its calenders (2), its body ironers (2), its collar machine, its goffering machine, its ironing tables, with eight or nine irons, and three or four skirt ironing boards. The advent of satisfactory electrically heated irons is to be hoped for, as there is no doubt whatever that gas irons smell; at any rate I have never found combustion so perfect that the nose is unable to detect the presence of unconsumed gas. The atmosphere of a laundry is bad in any circumstances, and no means should be left untried to improve it. Adjoining the ironing room is situated the drying room. There are many ways of drying clothes, but if a drying room is used it must be of large size; and it is convenient, and indeed far better for the workers, that the clothes should be hung on wheel horses and wheeled into the drying room, left there until they are dry, and wheeled out of the drying room to be unloaded. This saves the workers from hanging up and taking down the clothes singly in a very hot atmosphere. A separate drying room for blankets is required, as blankets cannot be subjected to the same heat as sheets and other linen goods. The last large room of the laundry is the packing room. Here space is essential. Round the walls should be ranged the receptacles. Each ward should have its compartment as well as each doctor, nurse, and maid. These compartments can be bought ready made in wire, though trellis-work of wood is quite good enough for the purpose. In the centre of the room there should be three or four large wooden tables. It is convenient also to have in addition a small airing room with shelves ranged round the walls, on which shirts, frocks, etc., can be thoroughly aired. The floors of both the ironing room and the packing room need not, of course, be of concrete, in fact it is better to have them of wood.

Though there is not the same need for separation in the sorting room, yet there is every reason why there should be a separate washhouse and a separate collecting room for the staff washing. A separate sorting room and a separate washhouse are not very difficult to arrange convenient to the common drying, ironing, and packing rooms. With regard to the foul and infectious linen of the hospital, it is necessary to have a small washhouse with a rotary washer, a hydro extractor, and steeping tanks. This is conveniently placed so that access to it from the disinfector is easy. An office placed so that the superintendent can conveniently overlook the washhouse and the ironing and packing rooms is essential and not difficult to arrange for. A store room for soap, etc., and the necessary lavatory accommodation must also be provided.

The staff required to work such a laundry includes a superintendent, who has received training in a commercial laundry or in a hospital laundry. Although we do not advocate commercial methods in a hospital laundry, yet an intelligent woman who has been trained in the art of washing will soon pick up the special requirements of hospital work. The matron under whom she works can soon make her realise the standard desired. She will require an assistant. It is advantageous also for her to be allowed to take pupils, who after a very short time become quite useful in the laundry. Six calender hands, six ironers, three washwomen, three packers, and one washman complete the staff. Although superintendents are often quite willing to work with nothing but women in a laundry, yet there is no doubt that there is an advantage in having a man. His superior strength and, if he is worth his salt, his readiness in an emergency are invaluable.

With regard to the question of the residence of the laundry workers. Local conditions must be taken into account in coming to any decision in this matter. Where a hospital is situated in the middle of the class from which it draws its workers, there is no difficulty in getting labour to come in day by day. Where a hospital is situated at a distance from the population, then not only is there difficulty in getting labour, but there is a hardship in asking women to come a distance to do a hard day's work—women sometimes so poor that even the saving of a tram fare, if there happens to be a tram, is of great consideration. The Factory Act, which now applies to hospital laundries also, has rather altered the situation. If the laundry girls are resident and under supervision after laundry hours, then the head superintendent and the assistant must have hours off during the daytime, and the cost is thereby increased. There is also difficulty with regard to the evenings out. In certain parts of England it is impossible to get girls to agree to, say, one or two evenings off in the week, and if they are allowed out after seven every evening the inevitable responsibility when things go wrong has to be faced. Unless the laundry girls are allowed their evenings off there is discontent no matter what arrangements you make for their amusement inside. There is, however, no doubt that the laundry block of a hospital should be self-contained during the day—that is, the laundry workers should have a separate and comfortable room in which to have their meals; and there is, I think, little doubt that a portion of the staff should be resident. The superintendent and the assistant superintendent must be resident, and the pupil and, say, four or five hands of proved respectability. To insist upon a resident staff would, of course, exclude married women, who are often the most responsible and the hardest workers.

Hospital Laundries.—The Ironing Room.

### The Wage Bill.

The cost of such a staff is:—

Head laundress, £40 per annum, with residence and uniform.

Assistant laundress, £25 per annum, with residence and uniform.

Pupil, £15 per annum, with residence and uniform.

Eighteen maids at wages ranging from 2s. 6d. to 10s. 6d. per week, with uniform—food being calculated at 5s. per week.

One washhouse man at 30s. per week with food.

The hours for female labour—7 a.m. to 7 p.m., with $1\frac{3}{4}$ hours off for meals.

The washhouse man—7 a.m. to 5.30 p.m., with $1\frac{1}{2}$ hours off for meals and half-day on Saturday from 1 p.m.

The age at which laundry girls can be engaged depends on physique, but not many are taken on before seventeen years of age. They may be allowed to go either through the whole curriculum—calenders, ironing, vester machine, collar machine, starching, hand ironing, and sorting and packing—which takes fifteen or sixteen months, or they may be allowed to do so much and then specialise—*e.g.* become ironers, packers, or washers. There is no difficulty in getting workers, as the advantage of an airy laundry, uniform, well-cooked meals, and regular work all the week through soon becomes known in the district.

### A Provincial Hospital with 430 Beds. The Actual Cost of Running its Laundry.

Subjoined is the actual cost of running a laundry for a hospital of 430 beds for one year. The only figures which are estimated are those which refer to the board of laundry hands, to the proportion of wages of engineers, stokers, etc., and to coals. A comparison with figures from other institutions should make due allowance for the difference in local rates of wages, in the local price of coals, gas, and water, some hospitals being supplied with this latter item free. The rest of the figures, of course, must stand comparison on their merits. The amount of work done is shown in a second table. The cost per dozen articles works out at about 8.8d.

Hospital Laundries.—The Packing Room.

### Washing Return for One Year.

| *Expenditure:* | £ | s. | d. |
|---|---|---|---|
| Wages of laundry hands ... ... | 532 | 16 | 7 |
| Board of laundry hands ... ... | 364 | 0 | 0 |
| Uniform of laundry hands ... ... | 14 | 11 | 0 |
| Insurance of laundry hands ... ... | 1 | 19 | $6\frac{1}{2}$ |
| Proportion of wages of engineer, stokers, etc. ... ... ... ... ... | 50 | 0 | 0 |
| Materials and sundries ... ... | 297 | 12 | $3\frac{3}{4}$ |
| Fuel, light, and power ... ... | 311 | 17 | 1 |
| Gas ... ... ... ... ... ... | 25 | 19 | 11 |
| Water ... ... ... ... ... ... | 57 | 13 | $9\frac{1}{2}$ |
| Repairs to machinery and plant ... | 69 | 8 | $9\frac{1}{4}$ |
| Insurance—Fire and boiler ... ... | 9 | 5 | 0 |
| Depreciation on buildings ... ... | 109 | 11 | 3 |
| Depreciation on machinery and boilers | 196 | 8 | $4\frac{1}{2}$ |
| Interest on capital ... ... ... ... | 420 | 0 | 0 |
| Rates ... ... ... ... ... ... | 3 | 0 | 0 |
| | £2,464 | 3 | $7\frac{1}{2}$ |

| | |
|---|---|
| Total pieces washed ... ... ... | 801,840 |
| Weekly average ... ... ... ... | 15,420 |
| Cost per 100 pieces ... ... ... | 6.147s. |

### A Week's Wash.

| | |
|---|---|
| *Personal:* | |
| Nurses ... ... ... ... ... | 3,700 |
| Maids ... ... ... ... ... | 550 |
| Residents ... ... ... ... ... | 250 |
| *Non-personal:* | |
| Household ... ... ... ... ... | 1,300 |
| Wards— | |
| Blankets ... ... ... ... ... | 320 |
| Sheets ... ... ... ... ... | 2,800 |
| Quilts ... ... ... ... ... | 200 |
| Other pieces ... ... ... ... ... | 4,000 |
| Departments ... ... ... ... ... | 2,300 |
| | 15,420 |

### Space in a Hospital Laundry.

The convenient sizes for the various rooms in a hospital laundry are as follows :—

| | |
|---|---|
| Collecting room ... ... ... | 30 ft. square. |
| Washhouse ... ... ... ... | 48 ft. square. |
| Ironing room ... ... ... | 50 by 40 ft. |
| Packing room ... ... ... | 25 by 40 ft. |
| Small airing room ... ... | 25 by 8 ft. |
| Drying room ... ... ... | 50 by 20 ft. |
| Blanket drying room ... ... | 50 by 20 ft. |
| Office ... ... ... ... | 20 by 12 ft. |
| Foul washhouse ... ... ... | 30 by 15 ft. |
| Store ... ... ... ... | 12 by 12 ft. |

## Figures and Tables

**Figure f1:**
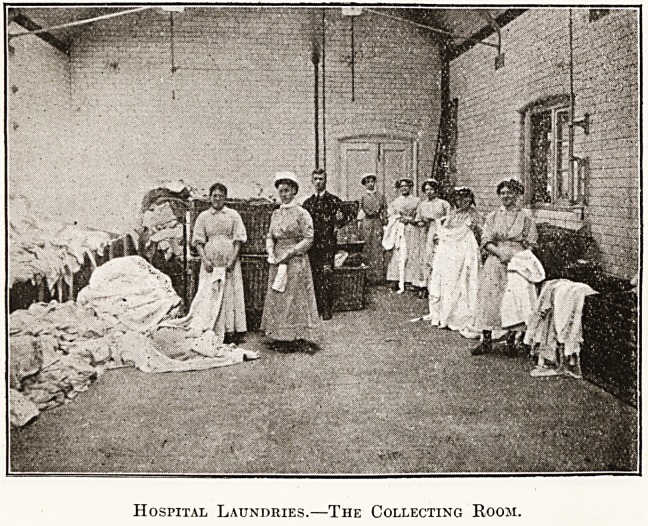


**Figure f2:**
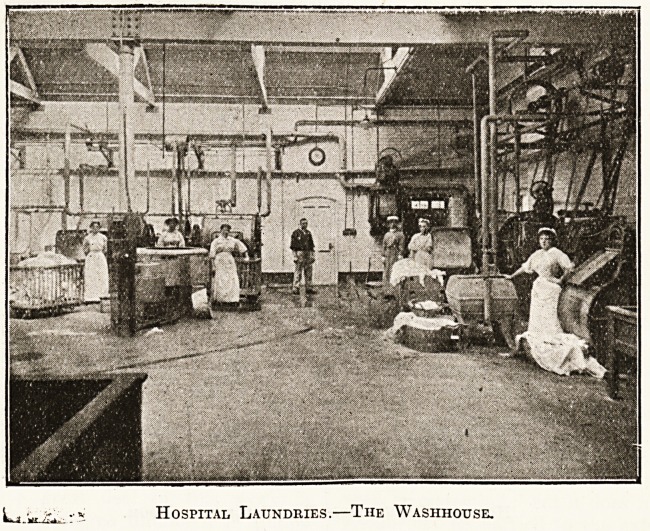


**Figure f3:**
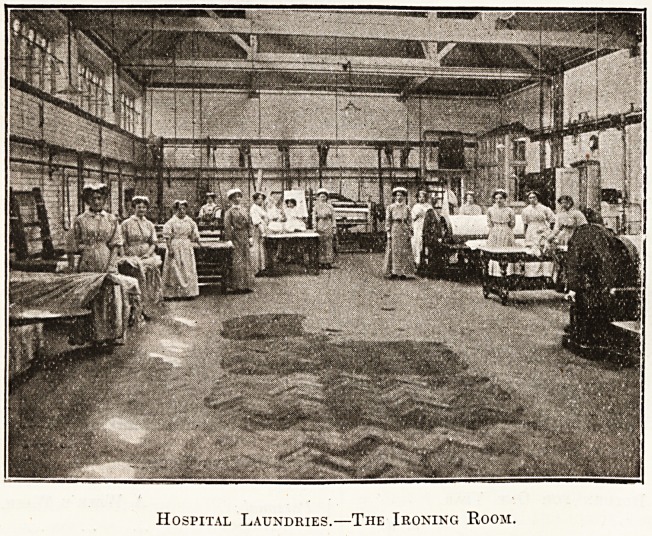


**Figure f4:**